# Association between uterine leiomyoma and fragmented QRS waves: a prospective case-control study

**DOI:** 10.1590/1806-9282.20231359

**Published:** 2024-05-03

**Authors:** Tugce Kacan Tatlici, Nurullah Cetin, Busra Korpe, Caner Kose, Vakkas Korkmaz

**Affiliations:** 1 University of Health Sciences, Etlik Zubeyde Hanim Women's Health Training and Research Hospital – Ankara, Turkey.; 2 Manisa Celal Bayar University, Department of Cardiology – Manisa, Turkey.; 3 Ankara Etlik City Hospital, Department of Gynecology and Obstetrics – Ankara, Turkey.; 4 Ankara Etlik City Hospital, Department of Gynecologic Oncology – Ankara, Turkey.

**Keywords:** Cardiovascular risk, Fibroids, Electrocardiography, Leiomyoma

## Abstract

**OBJECTIVE:**

The aim of this study was to evaluate the relationship between uterine leiomyoma and fragmented QRS, a non-invasive indicator of cardiovascular risk and myocardial ischemia, in women with uterine leiomyoma.

**METHODS:**

In this prospective case-control study, a total of 47 patients diagnosed with uterine leiomyoma (case group) and 47 healthy individuals without uterine leiomyoma (control group) who had undergone bilateral tubal ligation surgery were included. Various demographic, clinical, and laboratory parameters and the presence of fragmented QRS were recorded.

**RESULTS:**

The leiomyoma group showed significantly higher body mass index (27.46±2.18 vs. 25.9±2.87 kg/m^2^, p=0.005) and waist circumference (91.34±9.30 vs. 84.97±9.3 cm, p=0.001) compared with the control group. Uterine volumes were also significantly higher in the leiomyoma group (235.75±323.48 vs. 53.24±12.81 mm^3^, p<0.001). The presence of fragmented QRS was detected in 18.1% of the patients. Multiple regression analysis identified age, fasting blood glucose value, and the presence of fragmented QRS as independent risk factors for the presence of leiomyoma.

**CONCLUSION:**

This study provides valuable insights into the relationship between uterine leiomyoma and fragmented QRS. The presence of fragmented QRS was identified as an independent risk factor for the presence of leiomyoma. Further research is needed to better understand the underlying mechanisms connecting uterine leiomyoma and cardiovascular health.

## INTRODUCTION

Uterine leiomyomas, which are also known as fibroids or myomas, are the most common benign monoclonal tumors of the uterus, originating from uterine smooth muscle cells and fibroblasts^1^. They affect up to 80% of women by the age of 50 years and are associated with significant morbidity, including heavy menstrual bleeding, pelvic pain, infertility, and pregnancy complications^2^.

The pathophysiology of uterine leiomyomas is not fully understood, but there is evidence that genetic, epigenetic, hormonal, environmental, proinflammatory, angiogenic, and growth factors play a role^3–5^. Recent studies have shown that cardiometabolic risk factors contribute to the pathogenesis of uterine leiomyomas^6–10^.

Fragmented QRS (fQRS) is defined as the presence of an extra R wave (R’) or notching of the R or S wave or multiple R’ waves in two contiguous leads corresponding to a main coronary artery. fQRS is an ECG finding resulting from the heterogeneous activation of the ventricles due to myocardial scar. Studies have shown that fQRS is an indicator of poor prognosis in patients with acute myocardial infarction and a risk marker for arrhythmic events in cardiomyopathies^11^.

Investigating the relationship between fQRS and uterine leiomyomas is important^12^. By identifying the presence of fQRS in women with uterine leiomyomas, clinicians could potentially stratify patients according to their risk of cardiovascular events and provide appropriate treatment and follow-up. This could improve patient outcomes and quality of life.

To the best of our knowledge, there is no study investigating the relationship between uterine leiomyoma and fQRS in the literature. Common mechanisms may be shared in the development of uterine leiomyomas and atherosclerosis. Therefore, the aim of this study was to evaluate the relationship between uterine leiomyoma and the non-invasive and easily accessible indicator of cardiovascular risk and myocardial ischemia, fQRS, in women with uterine leiomyoma.

## METHODS

In this prospective case-control study, there were 47 patients diagnosed with uterine leiomyoma in the case group and 47 healthy individuals in the control group who had undergone surgery at a tertiary referral hospital between August 2021 and March 2022 for bilateral tubal ligation, in whom uterine leiomyoma was not detected on ultrasound. The necessary sample size was calculated using the G*Power 3.1.9.2 package program, based on the information obtained from the study by Zimmerman et al.^13^ with a power of 95%. Accordingly, the sufficient sample size was found to be 94 (n=47, 2 groups) when the error margin was 0.05 (alpha), the power of the study was 95% (power), and the effect size was 0.69. The study protocol was approved by the local ethics committee of the hospital.

Informed verbal consent was obtained from all patients who participated in the study by providing them with information about the research. Interviews with the patient and control groups were conducted face-to-face. Obstetric and gynecological history, including age, gravidity, parity, vaginal and cesarean delivery numbers, last menstrual period dates, menstrual patterns, contraceptive methods, previous surgeries, medication use, and medical background, was recorded. Patients were subjected to pelvic and physical examinations, and their heights and weights were measured to calculate their body mass index (BMI). Routine venous blood samples were taken from the antecubital area after a 10-h fast from preoperative patients. Fasting blood glucose, hemoglobin A1c (HbA1c), insulin, complete blood count, lipid profile, and biochemical parameters were analyzed from these samples. The “homeostasis model assessment-estimated insulin resistance (HOMA-IR)” formula was used to calculate insulin resistance: HOMA-IR=Fasting insulin Uiu-mL×FPG (mg/dL)/405. Preoperative ultrasound examinations were performed by the responsible researcher. Uterine leiomyomas were identified according to FIGO 2018, and their volumes were calculated using the ellipsoid formula (length×width×depth×0.52) by measuring the width, length, and depth of uterine leiomyomas and the uterus. The recorded data were entered into the patient form.

The ECONET Cardio M Plus ECG recording device was used to obtain a standard 12-lead surface ECG from all patients, and the ECGs were analyzed by an independent cardiologist who was blinded to the study. FQRS was defined as the presence of an RSR pattern and/or notching in the R and S waves in two adjacent leads corresponding to a major coronary artery region.

### Statistical analysis

The data were statistically analyzed using the SPSS (Statistical Package for the Social Science) Version 26 (SPSS, Chicago, IL, USA) program. The normal distribution of the data was evaluated by considering histogram and skewness-kurtosis values. Pearson's chi-square test and Fisher's exact test were used to compare the categorical variables between the groups. Categorical data were expressed as n (number) and percentages (%). Independent-samples t-test was used to compare the ordinal data of the groups, and ordinal data were presented as mean±standard deviation. Pearson correlation analysis was used to examine the correlations between variables. The relation between variables was also examined using univariate and multivariate regression analyses. The data were evaluated at a 95% confidence interval, and significance was accepted at p<0.05.

## RESULTS

The demographic and clinical characteristics of all patients are summarized in Table 1. The leiomyoma group had a significantly higher BMI (27.46±2.18 vs. 25.9±2.87 kg/m^2^, p=0.005) and waist circumference (91.34±9.30 vs. 84.97±9.3 cm, p=0.001) compared with the control group. The uterine volumes of the leiomyoma group were statistically higher than the control group (235.75±323.48 vs. 53.24±12.81 mm^3^, p<0.001) (Table 1). The comparison of laboratory and ECG parameters of the two groups is shown in Table 1.

**Table 1:** Demographic, laboratory, and electrocardiographic parameters of the patients and comparison of groups.

	Total patients (n=94)	Leiomyoma group (n=47)	Control group (n=47)	p-value
Age (years)	38.86±4.69	41.29±3.56	36.42±4.43	**<0.001**
Gravidity	3 (1–7)	3 (1–7)	4 (1–7)	0.234
Parity	3 (0–6)	2 (0–5)	3 (1–6)	0.126
BMI (kg/m^2^)	26.7±2.65	27.46±2.18	25.9±2.87	**0.005**
Abdominal circumference (cm)	88.15±9.81	91.34±9.30	84.97±9.35	**0.001**
Endometrial thickness (mm)	5.81±3.67	5.88±4.46	5.75±2.69	0.867
Uterine volume (mm^3^)	144.49±245.47	235.75±323.48	53.24±12.81	**<0.001**
fQRS positivity (n, %)	17 (18.1)	14 (29.8)	3 (6.4)	**0.006**
WBC (mm^3^)	8148.01±2812.26	8551.48±3149.11	7744.53±2395.83	0.165
Hemoglobin (g/dL)	12.08±1.61	11.45±1.47	12.7±1.51	**<0.001**
Hematocrit (%)	36.8±4.47	35.15±4.25	38.45±4.1	**<0.001**
Thrombocyte count (1/μL)	279.550±73.515	282.82±81.25	279.27±65.58	0.668
Glucose (mg/dL)	96.93±17.39	104.34±20.19	89.53±9.60	**<0.001**
HgA1c (%)	5.3±0.44	5.29±0.47	5.30±0.42	0.869
Insuline (mIU/mL)	12.60±1.55	13.60±1.94	11.61±10.62	0.538
ALT (U/L)	16.05±7.41	15.55±8.52	16.5±6.16	0.516
AST (U/L)	18.67±3.76	18.19±3.69	19.14±3.82	0.220
BUN (mg/dL)	9.71±3.014	8.66±2.68	10.76±2.98	**0.001**
Creatinine (mg/dL)	0.60±0.090	0.59±0.10	0.60±0.069	0.359
HDL (mg/dL)	51.17±13.03	48.82±13.36	53.51±12.39	0.082
LDL (mg/dL)	107.79±33.26	104.61±32.37	110.97±34.18	0.357
TG (mg/dL)	110.75±56.37	116.34±50.66	105.17±61.61	0.340
Cholesterol (mg/dL)	179.50±40.45	176.32±36.76	182.67±44.01	0.453
VLDL (mg/dL)	21.32±11.13	21.61±9.90	21.04±12.35	0.804
FSH (mlU/mL)	12.22±4.38	17.63±6.15	6.82±6.08	0.237
E2 (pg/mL)	74.072±16.65	90.51±45.77	77.63±23.42	0.141
LH (mlU/mL)	8.98±0.908	8.47±1.003	9.48±8.09	0.594
Heart rate (beat/min)	77.106±9.98	78.74±10.26	75.46±9.52	0.112
QT interval (ms)	373.17±24.14	373.12±28.78	373.21±18.72	0.986
QTc interval (ms)	423.117±27.31	428.06±31.67	418.17±21.34	0.080
HOMA-IR	3.24±3.34	3.85±6.9	2.63±2.61	0.257

ALT: alanine transaminase; AST: aspartate aminotransferase; BMI: body mass index; BUN: blood urea nitrogen; E2: estradiol; FSH: follicle-stimulating hormone; fQRS: fragmented QRS; HDL: high-density lipoprotein; HgA1c: hemoglobin A1c; LDL: low-density lipoprotein; LH: luteinizing hormone. Statistically significant p-values are indicated in bold.

When the leiomyoma characteristics of the study group were examined, a single leiomyoma was observed in 51.1% of the group, while the rate of those with more than three leiomyomas was 17%. When leiomyomas were evaluated according to the FIGO classification in our study, type 4 (27.6%) and type 5 (23.4%) myomas were more commonly observed, while type 1 (4.2%), type 2 (2.2%), and type 7 (2.2%) were less detected.

When the leiomyoma group was divided into two groups according to the presence of fQRS, there was no significant difference in terms of age, BMI, waist circumference, endometrial thickness, uterine and leiomyoma volume, and between the fQRS positive (n=14) and fQRS negative (n=33) groups (p>0.05) (Table 2).

**Table 2:** Comparison of demographic characteristics of groups according to the presence of fQRS in the myoma group.

	fQRS (-)	fQRS (+)	p-value
	(n=33)	(n=14)	
Age (years)	41.03±3.77	41.92±3.04	0.436
Gravidity	2.6±1.29	3.5±1.45	**0.043**
Parity	2.06±0.78	2.85±1.16	**0.009**
BMI (kg/m^2^)	27.2±2.33	27.8±1.79	0.427
Abdominal circumference (cm)	91.8±9.11	90.0±9.94	0.548
Endometrial thickness (mm)	5.77±4.51	6.14±4.52	0.798
Uterine volume (mm^3^)	259.7±79.91	179.3±100.54	0.442
Leiomyoma volume (mm^3^)	303.55±21.50	256.88±18.39	0.482

BMI: body mass index; fQRS: fragmented QRS. Statistically significant p-value are indicated in bold.

When we look at the correlation between the Leiomyoma properties and some variables, a negative correlation was observed between age and leiomyoma volume (r=-0.431, p=0.002). Positive correlations were observed between age and LDL-C (r=0.215, p=0.038), and age and the number of leiomyomas (r=0.359, p=0.013). Additionally, a positive correlation has been found between triglyceride levels and the number of leiomyomas (r=0.297, p=0.042).

When the relationship between variables and leiomyoma presence was evaluated by multiple regression analysis, age (odds ratio=0.688, p<0.001), fasting blood glucose value (odds ratio=0.893, p=0.003), and the presence of fQRS (odds ratio=11.350, p=0.032) were determined as independent risk factors. The relationship between variables and leiomyoma is presented in Table 3.

**Table 3:** Multivariate logistic regression analysis of the variables for leiomyoma.

	Odds ratio (95%CI)	p-value
Age (years)	0.688 (0.569–0.833)	**<0.001**
BMI (kg/m^2^)	0.813 (0.610–1.082)	0.156
Waist circumference (cm)	0.942 (0.862–1.029)	0.187
Glucose (mg/dL)	0.893 (0.829–0.963)	**0.003**
fQRS	11.350 (1.235–104.338)	**0.032**

BMI: body mass index; fQRS: fragmented QRS. Statistically significant p-values are indicated in bold.

## DISCUSSION

Among women in their reproductive years, uterine leiomyoma stands out as the most commonly encountered solid pelvic tumor. Utilizing two-dimensional saline contrast sonohysterography has demonstrated remarkable sensitivity in detecting endometrial polyps and submucosal uterine leiomyomas. This positions it as a potential primary diagnostic approach for investigating abnormal uterine bleeding in women^14^. According to the findings of Magalhaes et al., the levonorgestrel-releasing intrauterine system shows promise in managing abnormal uterine bleeding as well as uterine volume associated with leiomyomas and adenomyosis^15^. When addressing symptomatic patients, magnetic resonance-guided high-intensity focused ultrasound emerges as a safe and effective technique in treating uterine fibroids^16^.

In this study, the relationship between uterine leiomyoma and the presence of fQRS on electrocardiography was investigated in reproductive-aged women hospitalized preoperatively to hospital. Thus, it was aimed to predict subclinical cardiovascular disease risk by comparing the electrocardiograms of women with and without uterine leiomyoma.

A study by Korkmaz et al., investigated the cardiovascular risk of women with leiomyomas, carotid intima-media thickness (CIMT), insulin resistance, and lipid profile were used. BMI was significantly higher in the study group than in the control group^17^. A study by Uimari et al., investigated the relationship between uterine leiomyomas and cardiovascular risk, and BMI was found similar between the study and control groups^18^. In our study, BMI was found to be statistically significantly higher in the leiomyoma group than in the control group^17,18^.

A study by Tak et al., investigated the relationship between metabolic syndrome and premenopausal women, and the waist circumference was found to be 77 cm (72–83) in the myoma group and 76 cm (71–81) in the control group^19^. In our study, the waist circumference was significantly higher in the leiomyoma group (91.34±9.30 cm) than in the control group (84.97±9.35 cm). Uimari et al., showed that the risk of leiomyoma increased by 1 cm for each 1 cm increase in waist circumference (OR=1.02, 95%CI 1.00–1.04)^18^.

Tak et al., have shown that women with uterine leiomyoma have a higher likelihood of being diabetic compared with non-myomatous women and women with three or more leiomyomas have significantly higher fasting plasma glucose than those with one leiomyoma^19^. In our study, consistent with the literature, fasting glucose was significantly higher in the leiomyoma group.

The relationship between uterine leiomyomas and hyperlipidemia has been investigated in the literature. Tak et al., found higher low-density lipoprotein cholesterol (LDL-C) and lower high-density lipoprotein cholesterol (HDL-C) levels among women with uterine leiomyoma. They also found a positive correlation between the number of myomas and triglyceride levels and a negative correlation between the number of myomas and HDL-C levels^19^. Uimari et al., showed that for each 1 mmol/L increase in LDL-C, triglycerides, and total cholesterol levels, there is an increase in the risk of uterine leiomyoma^18^. Takeda et al., were unable to demonstrate a significant relationship between hypertriglyceridemia and uterine leiomyomas^20^.

Laughlin-Tommaso et al., aimed to investigate the risk of subclinical cardiovascular disease. Cardiovascular risk factors were found to be slightly more prevalent in women with myomas than in those without myomas. Hypertension was seen to be significantly higher in women with uterine leiomyoma, with an increasing difference at each follow-up. At baseline and 5 years of follow-up, there was no difference in the presence of coronary artery calcification (CAC) between women with and without myomas. After 10 years, the difference in the presence of CAC was significantly higher in women with myomas than in those without myomas (20.0 vs. 14.1%). At the end of the 5-year follow-up, the average CIMT was higher among women with uterine leiomyoma. The authors found that cardiovascular risk factors, especially BMI and hypertension, were higher in women with uterine leiomyomas than in those without myomas. All approximate measurements of subclinical disease, including CAC, mean CIMT, and mean LV mass, were higher in women with uterine leiomyomas. However, multivariate analyses did not show a relationship between leiomyomas and subclinical cardiovascular disease^21^. Aksoy et al., found a significant increase in CIMT in the myoma group compared with the control group^22^. Haan et al., reported that women with leiomyoma have a worse cardiovascular disease risk profile, including higher blood pressure, higher fasting plasma cholesterol and glucose levels, and more asymptomatic organ damage^23^.

Toraman et al., found fQRS to be a predictor of subclinical atherosclerosis in chronic kidney disease patients by looking at fQRS^24^. Fedulaev et al., found that the presence of fQRS in lateral derivations showed that it could be a non-invasive marker of severe coronary atherosclerosis^25^. Karabakan et al., found that fQRS could provide a comprehensive diagnostic approach for underlying cardiovascular diseases^26^. Aksu et al., found that fQRS was found to be associated with important cardiac electrical changes that could indicate an increased risk of atrial and ventricular arrhythmias^27^.

One of the limitations of our study was the absence of advanced cardiac evaluation methods such as echocardiography (ECHO). Another important limitation was that long-term follow-up of the evaluated cardiac and hormonal parameters was not performed because all included patients were in the preoperative period. However, despite these limitations, our study was prospective, and the study and control groups were homogeneous. To the best of our knowledge, this is the first study of its kind in the literature, and gynecological and cardiological parameters were independently evaluated by different investigators. Furthermore, the parameters examined in the study were based on objective tests, which are significant strengths of our study.

## CONCLUSION

It is shown that uterine leiomyomas and atherosclerosis development may share common mechanisms. In this study, we demonstrated a positive relationship between fQRS which is easily accessible and noninvasive diagnostic methods through ECG and uterine leiomyomas.
